# From pressure to tension: *a model of damaging inflation stress*

**DOI:** 10.1186/s13054-023-04675-4

**Published:** 2023-11-15

**Authors:** John J. Marini, Lauren T. Thornton, Patricia R. M. Rocco, Philip S. Crooke

**Affiliations:** 1 Department of Pulmonary and Critical Care Medicine, University of Minnesota, Minneapolis, St Paul, MN USA; 2 Laboratory of Pulmonary Investigation, Carlos Chagas Filho Institute of Biophysics, Federal University of Rio de Janeiro, Rio de Janeiro, Brazil; 3 Department of Mathematics, Vanderbilt University, Nashville, TN USA

**Keywords:** Acute respiratory distress syndrome, Ventilator-induced lung injury, Hyperinflation, Mathematical model, Elastic energy, Mechanical power, Tension

## Abstract

Although the stretch that generates ventilator-induced lung injury (VILI) occurs within the peripheral tissue that encloses the alveolar space, airway pressures and volumes monitor the gas within the *interior* core of the lung unit, not its cellular enclosure. Measured pressures (plateau pressure, positive end-expiratory pressure, and driving pressure) and tidal volumes paint a highly relevant but incomplete picture of forces that act on the lung tissues themselves. Convenient and clinically useful measures of the airspace, such as pressure and volume, neglect the partitioning of tidal elastic energy into the increments of tension and surface area that constitute actual stress and strain at the alveolar margins. More sharply focused determinants of VILI require estimates of absolute alveolar dimension and morphology and the lung’s unstressed volume at rest. We present a highly simplified but informative mathematical model that translates the radial energy of pressure and volume of the airspace into its surface energy components. In doing so it elaborates conceptual relationships that highlight the forces tending to cause end-tidal hyperinflation of aerated units within the ‘baby lung’ of acute respiratory distress syndrome (ARDS).

**Supplementary Information:**

The online version contains supplementary material available at 10.1186/s13054-023-04675-4.

## Background and objective

Ventilator-induced lung injury (VILI) relates to lung hyperinflation and/or repeated tidal cycles of intolerable mechanical ‘stretch’, which eventually fracture fragile stress-bearing elements or induce lung inflammation [1]. At today’s bedside, driving pressure (DP), plateau pressure (*P*_plat_), positive end-expiratory pressure (PEEP) and tidal volume (*V*_T_) are used to monitor that VILI risk. Energy, the area within the envelope that encloses inflation pressure and delivered volume, is required to inflate the lungs but may lead to damage; therefore, ‘power’, the product of frequency and inflation energy per cycle, has drawn increased attention as an integrating indicator of VILI risk [2–4]. Mechanical stress is a force-related variable that strains (displaces or deforms) tissue. Although both flow-resistive and non-resistive inflation pressures contribute to dynamic stress and strain, the conserved (so-called ‘elastic’ or static) pressures: *P*_plat_, PEEP and their difference, DP, are the measured pressure components of tidal energy commonly considered to stretch lung tissue [5].

**Fig. 1:**
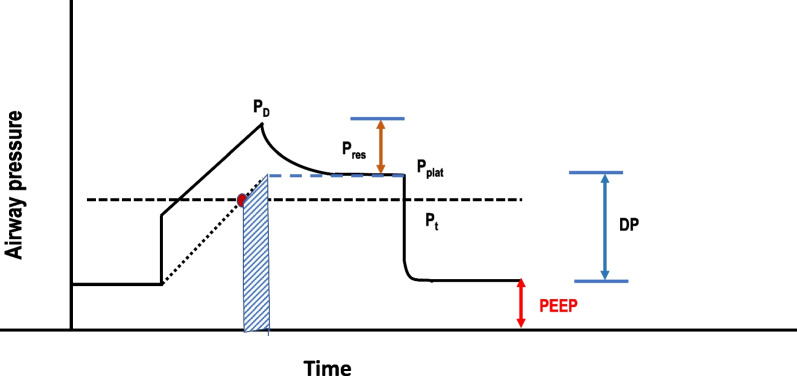
Damaging tidal energy (crosshatched area), as determined by an elastic pressure x volume area and demarcated by an elastic alveolar pressure threshold. Energy delivered at alveolar pressures above a pressure threshold potentially contributes to injury. P_D_ = peak airway pressure; Pres = non-conserved pressure dissipated through dynamic resistance; P_Plat_ = end-inspiratory elastic alveolar pressure; Pt = threshold pressure; DP = driving pressure

**Fig. 2:**
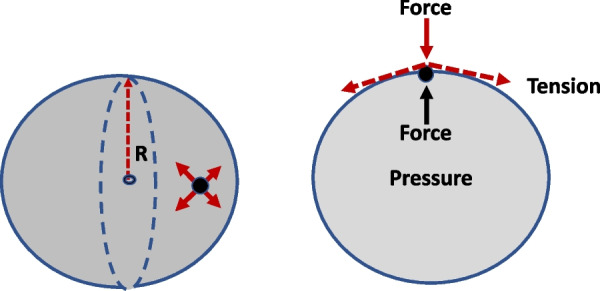
Force balance between outwardly directed interior pressure and inwardly directed tension at the surface of the sphere (T = PR/2) (left panel). The cut view (right panel) illustrates that the latter arise in part from the combined tangential force vectors within the curved SLM. In this illustration of geometrical relationships, the contribution of tissue recoil to the inward force vector is not considered. R = radius

Not all levels of elastic pressure or energy generate *excessive* power. More specifically, *damaging* energy recognizes a *threshold* for monitored pressure (Pt) below which delivered elastic energy can be viewed as ‘safe’ and above which the potential for hazardous overstretch may occur [6]. (Fig. 1) That threshold varies as a function of local transpulmonary pressures and tissue vulnerability to injurious stretch. Yet, even though gas pressure gives rise to them, VILI-inducing mechanical forces are actually generated at the periphery of the shell-like cellular membrane (SLM) that encloses the alveolar space through which gas pressures and volumes are applied and monitored. In concept, these ‘SLM’ stresses and strains relate more directly to changes of surface tension and area than to those of pressure and volume, respectively. (Fig. 2) If so, the tissue-rending force that acts on the cellular SLM and its matrix to initiate VILI may be better reflected by force/unit *length*, defined as *tension* than by pressure, which is force/unit *area *[7]. When considered at the alveolar boundary, neither measured pressures nor tidal volumes directly quantify tidal tissue strains defined as the incremental expansion of surface area. The same might be said of clinically measured energy, power, and each of their component variables measured from *within* the alveolar airspace.

While the concept of ‘damaging energy’ as determined by crossing of a pressure-defined stress threshold remains highly relevant, the primary purpose of the current work is to refine and complement our previous model of damaging energy [8] to draw a closer connection between the variables routinely measured at the bedside (*V*_T_, *P*_plat_, PEEP, DP) and the stresses and strains that actually result from them at the alveolar boundary. Because stretch occurs at the periphery of the alveolar unit and not within the airspace core itself, we propose that, in theory: (1) *Tension* is the relevant force per unit length that stretches the SLM to strain cellular elements; and (2) The partitioning of elastic energy into tension and surface area helps understand how well measured pressure, volume, and their product approximate ventilatory stress, damaging energy per inflation cycle, and hazardous power [6, 8]. We emphasize that these are mathematical relationships and though relevant, do not intend to mimic the complexity of the true biological state.

## Model development

### Radial and surface energy

Details that underpin our mathematical model are presented in the online Additional file 1: (Parts 1–4). The first step in developing a conceptually informative yet clinically relatable model from variables routinely measured at the bedside requires important assumptions and simplifications. Prominent among these in our model is to represent the alveolar compartment of the lung considered at any scale as a single expandable, thin-walled and balloon-like hollow *sphere* of radius (*R*) with uniform inflation properties. These properties are characterized by a constant compliance (*C*) applicable to all measured increments of pressure and volume. In actuality, lung units are irregularly shaped and interdependent. Moreover, at low lung volumes even an open unit may unfold as its pressure increases, rather than recoil to store elastic energy [9, 10]. Although such basic geometry is clearly an oversimplification, we note that the shape of the individual alveolus does, in fact, become more spherical as volume increases toward its maximum [9], a process that reduces the ‘corner irregularities’ where stress is focused [9, 11]. While all forms of non-dissipated energy input to the sphere—the conserved or ‘elastic’ energy—must sum to the same value, the *partitioning* of the components of that total energy can vary independently. [Derivation of this equivalence is provided in the Additional file 1: Part 1]. Using a highly simplified spherical model allows translation of total *radial* elastic energy, (a measurable pressure (*P*)—volume (*V*) quantity), into *surface* energy, (a circumferential tension (*T*)—surface area (*A*_s_) quantity). This radial to surface energy conversion permits estimation of the relevant stresses and strains within the cellular SLM.

The incremental elastic energy needed for inflation during tidal inflation is stored under tension within the expanding margin of the hollow sphere where the cellular elements reside; conceptually, therefore, increments of circumferential tension (*T*) and surface area (*A*_s_) determine the tissue stretch that stimulates VILI. In such a basic model, the stressing variable relevant to the alveolar tissue is *tension* (*T*), not simply the directly accessible intracavity *pressure* measured within the alveolus and ventilator circuitry. Technically, T is not formally defined as stress because *T* is a force/length, whereas stress is force/area [7]. Tension at the surface is generated by forces developed tangential to the radius (*R*) of the sphere. In accordance with the ‘law of LaPlace’ for such thin-walled expandable spheres: *T* = PR/2, where P is the sphere’s internally measured pressure [12] (Fig. 2). Consequently, the greater the diameter of an individual spherical lung unit, the greater its SLM tension for a given pressure. This relationship applies to spheres of any dimension—whether large or small. Importantly, unlike the equivalence of input elastic *energy* to increments of surface elastic energy, the subcomponents of these identical energies differ. Yet, under static, no-flow conditions, radial *forces* resulting from *P* must be exactly counterbalanced by the net inward vector of non-radial (tangentially orthogonal) surface *forces* that arise from circumferential tension–what are often known as ‘hoop stresses’ [13] (Fig. 2). This mandate to balance the outward force of a given pressure with the inwardly directed force generated by circumferential tension holds true whatever the sphere’s radius. The smaller the sphere’s radius and sharper its surface curvature, the greater the leverage of tension to generate that inward force vector (Fig. 2). Reduced leverage angle for generating its inward force vector requires tension to rise disproportionately to area as the alveolus distends.

With the difference between *T* and *P* as well as the equivalence of surface energy and radial energy in mind, it follows that *strain* imposed by inflation on the cellular SLM is not the simple relationship of *volume* increment (∆V) to resting volume (*V* = 4π*R*^3^/3), but a more direct correlate of the incremental change in the surface *area of the SLM* (*A*_s_). This SLM area is A_s_ = 4πR^2^. Because the relationship of T and A_S_ quantifies the incremental elastic energy stored in the SLM during inflation, alternative estimates of energy input integrating P and V or T and A_s_ are identical, but in these estimates, ∆*V* ≠ ∆*A*_s_. (Derivation of their quantitative relationship is provided in the Additional file 1: Parts 1 and 2.) As already noted, assuming perfect efficiency of the translation of non-dissipated airspace energy to surface elastic energy, ∆T must differ from ∆P during expansion to maintain equivalence between input radial and SLM-stored elastic energies. How they differ relates to R and its incremental change: $\Delta T=\frac{1}{2}R\Delta P+\frac{1}{2}P\Delta R$.

### Role of unstressed volume (FRC) in components of surface elastic energy

For a sphere, the two key variables defining T are P and R, the first of which can be directly measured in clinical practice and the other only estimated. (The Additional file 1: part 3 outlines the rationale underpinning that estimation.) In broad outline, the value of R for our lung model derives from its absolute volume expressed in clinical terms: *V* = FRC + PEEPxC + *V*_T._ With the resting volume, or functional residual capacity, FRC, estimated as described in Additional file 1: part 3, R of a sphere at either the macro (lung) or micro (subunit) scales is determined from its corresponding absolute volume *V*_A_ as: *R* = (3*V*_A_/4π)^1/3^. This R can then be used to estimate the corresponding ‘SLM’ tensions (T = PR/2) and ‘SLM’ areas, A_s_ = 4πR^2^ of interest.

In a clinical context, *V*_A_ at end-expiration equals its unstressed FRC plus any PEEP-related volume increment (*V*_PEEP_). The *V*_A_ at end-inspiration is the sum of FRC, *V*_PEEP_ and *V*_T_. In practice, the FRC of an ARDS ‘baby lung’ typically goes unmeasured but bears an inverse relationship to the degree of ARDS severity [14]. Whatever the severity, the baby lung of ARDS is assumed by convention to be comprised of a number (*n*) of normally compliant individual alveolar subunits [14, 15]. Under this assumption, measured tidal compliance (*C*_obs_) is a direct function of the *number* of quasi-normal subunits, not of subunit stiffness. We note here, however, that while their expansion properties may be similar, the vulnerability of a heathy lung unit to stretching injury is likely much less than a pre-injured unit. Moreover, immersed in a heterogeneous mechanical environment, the latter are also exposed to the amplified forces of stress focusing. It follows that the unmeasured FRC is highly VILI-relevant, as it correlates inversely with individual subunit diameter at end-inflation and the resulting stretch that occurs as the n units of the baby lung accommodate and share the tidal volume (*V*_T_). Though unmeasured in the individual patient, a reasonable (though imprecise) estimate of the baby lung’s resting and unstressed volume without any PEEP applied (*V*_rest_) might be: *V*_rest_ = (*C*_obs_/C_norm_) $\times$ FRC_pred_, where predicted normal FRC (FRC_pred_) is a known function of age, gender, and lean body mass during health [16, 17]. The *C*_obs_ of the respiratory system is directly calculated from monitored tidal pressures and tidal volume. Normal compliance (*C*_norm_) varies modestly with body position but generally ranges from 80–100 ml/cmH_2_O [17].

### Comparison within and between baby lungs of different capacities

**Fig. 3:**
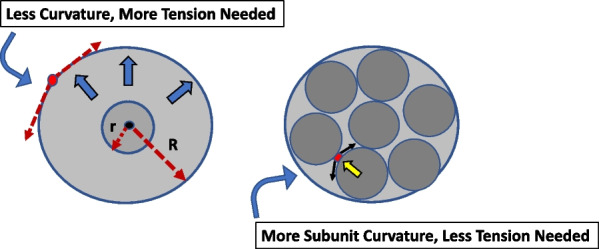
Comparison of wall tension of two spheres with different composition but the same pressure and cumulative volume. Left panel: Tension increases as a single unit increases its volume from radius r to R, in accordance with the LaPlace equation. Right panel: In a sphere comprised of multiple such subunits, the tighter curvature of each subunit (each of radius r) as opposed to that of their encapsulating SLM (with radius R), helps subunit force balance to be achieved with less wall tension

The LaPlace equation dictates that expansion of any given sphere increases its wall tension. For a spherical structure of volume (*V*), however, the tension (*T*) that develops within its spherical subunits will depend upon the number (n) of such units that comprise their collective volume (*V*) (Additional file 1: Part 2). We note that when expressed in the same numerical units, a larger sphere that contains the same net volume as a collection of smaller subunits will have a SLM tension that exceeds the wall tensions of its constituent subunits (Fig. 3). More specifically, a simple calculation indicates that for two *non-communicating* collections (A and B) of identical cumulative volume containing *n*_A_ and *n*_B_ numbers of subunits, respectively:

$${\mathrm{TB }=\mathrm{ TA }/({n}_{\mathrm{B}}/{n}_{\mathrm{A}})}^{1/3}.$$

(Additional file 1: 2)In other words, the same absolute volume contained in a separate sphere comprised of fewer units will have higher SLM subunit tension; therefore, increasing the capacity (subunit numbers) of the hypothetical baby lung will reduce tensions in the individual subunits that comprise it.

### Accounting for wall thickness and subunit stiffness

**Fig. 4:**
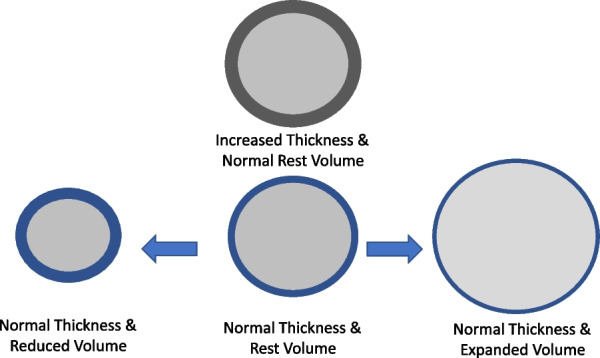
Effects of thickness on spherical wall tension. According to the modified LaPlace equation (T = PR/2z), the thicker of two spheres with the same volume will experience less wall tension from the same internal pressure (center). In a sphere whose wall has a thickness comprised of a fixed quantity of expandable biomaterial, expansion decreases (thins) relative thickness, helping augment wall tension (right). Conversely, lower volumes increase relative wall thickness, diminishing wall tension (left)

To this point, the SLM of the sphere has been considered of negligible thickness. But in fact, alveolar walls *do* have thickness comparable to their radii. Discounting any change of capillary blood volume, a biological alveolar ‘sphere’ would have a fixed quantity of tissue that thins with stretching. In this setting, the *modified* LaPlace equation applies: *T* = PR/2z, where z represents wall thickness. Starting from a SLM with an unchanging amount of cellular tissue, wall thickness will increase as the sphere contracts and decrease with its expansion. (Fig. 4) Therefore, at high volumes, *T* is increased not only by higher P and R, but to some extent by lower *z* values. Along this line, within the same lung, modeled alveoli of smaller radii (e.g., gravitationally dependent) should have lower wall tensions than non-dependent ones at the same pressure, due at least in part to their greater wall thickness. For the same thickness and internal volume, a stiffer alveolar unit or one amidst non-inflating tissues that buttress it (e.g., in late-stage ARDS) would experience less tension and stretch less in response to a pressure increase. For these, the pressures that demarcate the threshold for damaging energy would theoretically increase.

### Model predictions

Allowing for its basic assumptions, this simple model yields predictions of potential interest to questions that surround VILI avoidance. These include:

1. Within a given baby lung, SLM tension of its constituent lung units increases disproportionately to measured pressure, assuming C remains unchanged.
2. For set values of V_T_ and PEEP, end-inspiratory SLM tension (PR/2), the rending (or ‘ripping’) force per unit length, relates inversely to FRC at zero PEEP.
3. Strain resulting from *non-dissipated* (‘elastic’) energy during inflation from R_1_ to R_2_ may differ from that estimated from measured tidal volumes and pressures because ∆ surface area is not identical to ∆ volume (Additional file 1: Part 4):
  $$\frac{\Delta A}{\Delta V}=[3\left({{R}_{2}}^{2}-{{R}_{1}}^{2}\right)]/({{R}_{2}}^{3}-{{R}_{1}}^{3})$$
  Consequently, tension rises in direct relation to baby lung *unit volume*, but hazardous energy causing excessive strain (stretch) relates more closely to relative changes in *surface area* than to those in volume. What’s more, as volume increases, the input energy causes T (the stressing factor of SLM energy) to rise faster than A_S_ (the strain factor), thereby altering the pressure threshold for damaging energy.
4. Lung unit stiffness, SLM thickness, or smaller unit dimensions lessen the tension developed in response to a given pressure increment. Simultaneously, the discrepancy narrows between the variables indicating stress (pressure vs. tension).
5. As the cellular SLM thins during expansion, increments of damaging energy accentuate the tendencies for alveolar coalescence and cystic transformation, because ∆A_S_ and ∆T both rise for a given ∆P. Thinning of the SLM accentuates this tendency.

## Clinical relevance

This highly simplified modeling exercise is not intended for direct and quantitative clinical translation. In opening a discussion of tension based on single isolated spherical structures, we understand that the architecture and micromechanics of the injured lung are far more complex. Nonetheless, consideration of the shared fundamental physical principles of the model may help instruct bedside respiratory support. At the alveolar periphery, the damaging portion of ∆ PV energy translates into increments of tension and area, which logically are the actual generators and correlates of hazardous stress and strain. Tidal increments of energy weigh progressively toward its SLM tension component. We note here that rising tension is not always detrimental; until a damage threshold is crossed [6, 8], higher tension may be well tolerated or help in the recruitment of unstable units [11]. Once beyond that threshold, however, unnecessary volume increments are to be avoided. Minimizing disparities of regional volumes and tensions, as by prone positioning, is also advisable.

Awareness of lung unit hyperinflation during ARDS has received increased recent attention [18–20]. Unmeasured end-expiratory volume *per aerated lung unit* is a key determinant of cellular stretch during V_T_ delivery. In that context, a sharper focus on disproportionately rising SLM tension underscores the importance of avoiding end-tidal overdistention by lessening mechanical heterogeneity and avoidance of excessive tidal volumes or unwarranted high PEEP. Even when recommended guidelines are followed, end-tidal overstretching of the small ‘baby lung’ may help account for the ‘paradoxical responses’ to chest wall compression and for more frequent barotrauma in ARDS-associated Covid-19 pneumonia [21]. Localized overexpansion increases vascular resistance, redirects blood flow, and disrupts optimal ventilation/perfusion matching. More VILI-relevant, escalating tension coupled with SLM thinning during expansion increases the risk of disruption and of coalescence of multiple small units into fewer but larger ones containing the same collective volume, raising tension at the airspace periphery (Fig. 3). This tendency to merge alveolar subunits accentuates when the stress-bearing extracellular matrix elements and microstructures have been degraded by inflammation, encouraging rapid formation of what has been termed ‘tension cysts’ [22]. Rarely, these high airspace and vascular stresses, acting together, have the potential to disrupt the gas to blood barrier and generate systemic gas emboli. [23].

While extensive subunit overdistention must be avoided, our model also underlines why recruitment at safe plateau pressures is desirable, as adding functional units not only reduces driving pressure but also reduces the end-tidal alveolar tension developed during breath delivery. Conversely, advancing disease or VILI reduce the number of aerated units, shrinking the ‘baby lung’, thereby raising subunit surface energy, encouraging end-tidal hyperinflation, and predisposing to cystic transformation [21, 24, 25].

## Model limitations

While perhaps conceptually valid in its basic premise, such a simplified mathematical model clearly has limited correspondence with the complex geometry that characterizes the actual biological environment of the injured lung. In our model’s unmodified form, La Place’s ‘law’ for thin-walled *spheres* must apply to all compartment sizes, and wall thickness is not considered. In vivo, however, neither the integrated lung and nor its constituent units are negligibly thin, mechanically independent identical spheres exposed to a single transpulmonary pressure. In actuality, the latter vary locally in accordance with their immediate local environments, causing regional differences of resting subunit volumes [10]. Moreover, alveolar walls are relatively thick in relation to their radii, and biological lung unit contours are irregular and interdependent, with highly variable topography of stress-focused corners and interfaces [9, 10]. Importantly, the assumption of quasi-normal specific compliance of all aerated baby lung subunits, while perhaps initially reasonable, is open to serious question in the later stages of advancing ARDS, when organizing inflammation and fibrotic processes are underway. In every phase of ARDS, constancy of the pressure–volume relationship (unchanging subunit compliance) seems unlikely to apply at the extremes of the inflation range. Finally, the perception itself that individual alveoli alter their radii after opening remains somewhat controversial [10], as does the structure that actually expands—alveolar ducts or sacs (10). While unfolding that recruits SLM may occur at low dimensions, once unfolded, tension rises as a direct function of pressure at greater dimensions, whatever the shape.

## Summary and conclusion

The stretch that generates VILI occurs in the cellular enclosure (SLM) of the alveolar space, while airway pressures and volumes monitor the *interior* core. Though clinically useful, P_plat_, DP and V_T_ paint an incomplete picture of SLM forces and neglect the partitioning of applied tidal energy into the increments of tension and change of surface area occurring at the periphery. The absolute lung unit volume, influenced by resting FRC as well as by PEEP and V_T_, influences SLM tension. The resulting forces applied to the SLM are more tangential than radial, and these directly influence the actual stresses and strains experienced by the alveolar cells at risk for VILI, especially at points of discordant compliance–stress-focused interfaces. Because the radius of the lung subunit is a fundamental component of tension, reduction in their number promotes the tendency for end-tidal hyperinflation and eventual microcyst formation. For these aspects of VILI, the ‘elastic’ pressure and tidal volume products that currently define damaging energy (DE) and power (f x DE) need further refinement to consider the tension x area product that comprises elastic energy applied and stored at the alveolar boundary.

All authors reviewed and agreed with the final version of this manuscript.

### Supplementary Information

**Additional file 1**. Rationale and detailing of the mathematical model.

## Abbreviations

ARDS: Acute respiratory distress syndrome
*A*_S_: Surface area
*C*: Compliance
*C*_norm_: Predicted compliance
*C*_obs_: Observed compliance
DE: Damaging energy
*F*: Force
DP: Driving pressure (*P*_plat_ minus PEEP)
FRC: Functional residual capacity (unstressed resting volume)
*N*: Number
*P*: Measured pressure
PEEP: Positive end-expiratory pressure
*P*_plat_: Plateau pressure (end-inspiratory elastic pressure)
*P*_t_: Threshold pressure for damage
*R*: Sphere radius
*T*: Tension at the sphere’s surface
SLM: Shell-like membrane enclosing the alveolar sphere
*V*: Volume
*V*_A_: Absolute (total) volume
VILI: Ventilation-induced lung injury
*V*_Peep_: Volume increment due to PEEP
*V*_Rest_: Volume at rest (at FRC)
*V*_T_: Tidal volume
