# Supplementary material for: From pressure to tension: a model of damaging inflation stress
Source: Crit Care. 2023 Nov 15;27:441. doi: 10.1186/s13054-023-04675-4 (PMC10652628; doi:10.1186/s13054-023-04675-4)
Supplement: Supplementary file 1 — Additional file 1. Rationale and detailing of the mathematical model. [file 13054_2023_4675_MOESM1_ESM.docx]

**Supplement Integrated**

**Part 1. Free (elastic) Energy of an Inflating Sphere**

We examine the free (elastic) energy added to the dynamic system of inflating an elastic spherical balloon-like sphere by increasing its internal pressure. We will perform the calculation using two approaches: (1) pressure-volume and (2) tension-area.

**1A. Pressure-Volume**

The Helmholtz free energy (or just free energy) from the first law of thermodynamics in differential form is:

$$df=sd\theta+PdV$$

where *f* is free energy, *s* is the entropy of the system, $\theta$ is the temperature, $P$is the pressure, and $V$is the volume. We assume that the system is at constant temperature so that $df=PdV$. Integrating and assuming that we are increasing the free energy of the expanding sphere, we have:

$$f=\int PdV$$

We calculate the change in free energy as the balloon is inflated from a radius of $R_{0}$ to a radius of $R$. Then the change in free energy is given by the formula:

$$\Delta f=\int_{R_{0}}^{R} P(r)\cdot\left( 4\pi r^{2} \right)dr$$

where $V\left( r \right)=\frac{4}{3}\pi r^{3}$ and $dV=4\pi r^{2}dr$.

We assume that the pressure within the sphere is related to its volume by the formula: $P\left( r \right)=\frac{V(r)}{C}=\frac{\frac{4}{3}\pi r^{3}}{C}$ where $C$ is the compliance. We assume that this compliance remains constant over the range of pressures in the inflation. Hence,

$$\Delta f=\int_{R_{0}}^{R} \frac{\frac{4}{3}\pi r^{3}}{C}\cdot\left( 4\pi r^{2} \right)dr=\frac{16\pi^{2}}{3C}\int_{R_{0}}^{R} r^{5}dr=\frac{8\pi^{2}}{9C}\left( R^{6}-R_{0}^{6} \right)$$

**1B. Tension-Area**

The free energy change in differential form in terms of surface tension and surface area is:

$$df=TdA$$

where *f* is free energy, $T$is the tension, and $A$is the surface area. Integrating and assuming that we are increasing the free energy of the expandable sphere, we have:

$$f=\int TdA$$

As in the pressure-volume calculation, we calculate the change in free energy as the sphere inflates from a radius of $R_{0}$ to a radius of $R$. Here $A\left( r \right)=4\pi r^{2}$ and we have:

$$\Delta f=\int_{R_{0}}^{R} T(r)\cdot\left( 8\pi r \right)dr$$

We assume that the surface tension is given by $T\left( r \right)=\frac{P(r)r}{2}=\frac{\frac{4}{3}\pi r^{4}}{2C}$ where $C$ is the constant compliance. Hence,

$$\Delta f=\int_{R_{0}}^{R} \frac{\frac{4}{3}\pi r^{4}}{2C}\cdot\left( 8\pi r \right)dr=\frac{16}{3C}\int_{R_{0}}^{R} r^{5}dr=\frac{8}{9C}\left( R^{6}-R_{0}^{6} \right)$$

which is the same value for the change of free energy that we observed in the pressure-volume calculation.

The tension (T) is a force tangential to the surface of the sphere. Consider a point on the surface of the sphere and consider the tangent plane to the surface at that point. In the tangent plane consider an orthogonal coordinate system with the coordinate axes directed in the circumferential and longitudinal directions of the sphere:


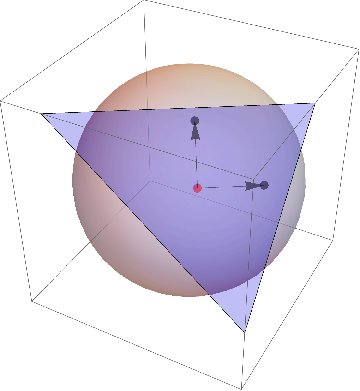


The change in free energy by these “hoop forces” is given by the above formula. Because of symmetry, every point on the sphere’s surface undergoes the same change of free energy. If the sphere were not homogeneous, then some points would fail (tear) when the threshold energy was achieved.

**Part 2. Comparing Tensions in Uni- & Multi-Compartment Models**

**2A: Dependence of Tension on Driving Pressure for a Single Compartment Lung**

From Laplace’s equation, we have:

$$T=\frac{1}{2}PR$$

where $P$ is the pressure with the spherical balloon and $R$ is the radius. The pressure is composed of different components:

$$P=P_{rest}+PEEP+P_{applied}$$

The applied pressure is the driving pressure $\left( DP=\frac{V_{T}}{C} \right)$. The radius is the solution of the equation:

$$FRC+C\cdot PEEP+V_{T}=\frac{4}{3}\pi R^{3}$$

or

$$R=\sqrt[3]{\frac{3}{4\pi}\left( FRC+C\cdot PEEP+V_{T} \right)}$$

Hence,

$$T=\left( \frac{FRC}{C}+PEEP+DP \right)\cdot\sqrt[3]{\frac{3}{32\pi}\left( FRC+C\cdot PEEP+C\cdot DP \right)}$$

Hence, the tension is a nonlinear function that depends on the PEEP and driving pressure. Furthermore, tension is an increasing function of PEEP and/or driving pressure. If $PEEP=0$ and $FRC\ll1$, then

$$T\approx DP\cdot\sqrt[3]{\frac{3C}{32\pi}DP}=\sqrt[3]{\frac{3C}{32\pi}}\left( DP \right)^{4/3}$$

This relationship implies that the tension increases with driving pressure like: T $\approx constant\cdot\left( DP \right)^{4/3}$ , where the constant is: $\sqrt[3]{\frac{3C}{32\pi}}$i.e., T rises faster than linearly with DP. One might ask: How does the total pressure within the balloon depend on driving pressure? By construction, that pressure is given by the expression:

$$P=\frac{FRC}{C}+PEEP+DP$$

i.e., the pressure increases at the same rate as $DP$. Hence, tension increases faster than pressure with increasing driving pressure. We note that since the plateau pressure is defined as $P_{p;ateau}=PEEP+DP$, the same relationship between tension and driving pressure holds for tension and plateau pressure.

**2B**: **A Model for *Independent* Multi-Alveolar Compartments**

In this section, we examine the surface tension in two contrasting models: (1) single spherical compartment and (2) a model composed of several spherical compartments. In particular, suppose we have two models: (1) a single spherical compartment with radius *R* and hence volume $V=\frac{4}{3}\pi R^{3}$ and (2) and a compartment composed of *n* spheres of the same radius *r* with the same total volume: $V=n\left( \frac{4}{3}\pi r^{3} \right)$. Hence, $r=\frac{R}{\sqrt[3]{n}}$ . For example, if $n=1000$, then $r=\frac{R}{10}$ . Let $T_{R}$ denote the surface tension of larger compartment and $T_{r}$ the surface tension in each of the individual smaller compartments. By Laplace’s formula, we have

$$T_{R}=\frac{2}{3C_{R}}\pi R^{4}$$

$$T_{r}=\frac{2}{3C_{r}}\pi r^{4}$$

where $C_{R}$ and $C_{r}$ denote the compliances of the single larger compartment and a single smaller compartment, respectively. Here we assume that the compliances are constant over the range of volumes used to inflate each model. The compliances are related by the following equation:

$$\frac{1}{C_{R}}=\sum_{i=1}^{n} \frac{1}{C_{r}}=\frac{n}{C_{r}} \Longrightarrow C_{R}=\frac{C_{r}}{n}$$

It follows that

$$T_{R}=\frac{2}{3C_{R}}\pi R^{4}=\frac{2}{3\left( \frac{C_{r}}{n} \right)}\pi R^{4}=\frac{2\pi nR^{4}}{3C_{r}}$$

and

$$T_{r}=\frac{2}{3C_{r}}\pi\left( \frac{R}{\sqrt[3]{n}} \right)^{4}$$

We can then form the ratio of the two tensions:

$$\frac{T_{r}}{T_{R}}=\frac{\frac{2}{3C_{r}}\pi\left( \frac{R}{\sqrt[3]{n}} \right)^{4}}{\frac{2\pi nR^{4}}{3C_{r}}}=\frac{1}{n^{4/3}}$$

or

$$T_{r}=\frac{T_{R}}{n^{7/3}}$$

This relationship between the tensions in the two models implies that the tension in the small compartments is reduced by $\mathcal{O(}n^{\frac{7}{3}})$ and not by $\mathcal{O(}n)$ as one might guess.

**2C**. **A Model for *Linked* Multi-Alveolar Compartments**

Suppose a lung system is composed of two multi-alveolar compartments each having a different number of identical alveoli which are spheres of radius $R$. The larger of the two compartments we call the *normal* lung and the smaller one the *baby* lung. In particular, suppose the baby lung is composed of $N_{babv}$identical alveoli and the normal part of the lung is composed of $N_{normal}$ alveoli of the same shape and composition as the baby lung. The overall compliances of the two can be computed from the compliance of the individual alveolus ($C_{alveolus}$):

$$\frac{!}{C_{baby}}=\sum_{i=1}^{N_{baby}} \frac{1}{C_{alvoelus}}=\frac{N_{babv}}{C_{alvoelus}}$$

$$\frac{!}{C_{normal}}=\sum_{i=1}^{N_{normal}} \frac{1}{C_{alveolus}}=\frac{N_{normal}}{C_{alvoelus}}$$

$$\Longrightarrow C_{baby}=\frac{C_{alveolus}}{N_{babv}}, C_{normal}=\frac{C_{alveolus}}{N_{normal}}$$

$$\Longrightarrow\frac{C_{baby}}{C_{normal}}=\frac{N_{normal}}{N_{babv}} or C_{baby}=\left( \frac{N_{normal}}{N_{babv}} \right)C_{normal}$$

If both compartments are connected by a common airway, suppose a volume $V$ is delivered uniformly among the alveoli in each compartment. We next calculate the tension in each alveolus for each compartment. By assumption, the volume in each alveolus will depend on which compartment that they belong. Let $\alpha$ be a partitioning parameter such that

$$V_{normal}=\left( \frac{N_{normal}}{N_{normal}+N_{babv}} \right)V$$

and

$$V_{baby}=\left( \frac{N_{babv}}{N_{normal}+N_{babv}} \right)V$$

With this partitioning of volume between the two compartments, we can calculate the volume of each alveolus, depending on which compartment it belongs.

Using LaPlace’s approximation for the tension in the normal lung alveolar and in the baby lung alveolar, we have:

$$T_{normal}=\frac{PR}{2}=\frac{\left( \frac{V_{normal}}{N_{normal}} \right)R}{{2C}_{alveolar}}=\frac{\left( \frac{1}{N_{normal}+N_{babv}} \right)VR}{{2C}_{alveolar}}$$

$$T_{baby}=\frac{PR}{2}=\frac{\left( \frac{V_{baby}}{N_{babv}} \right)R}{{2C}_{alveolar}}=\frac{\left( \frac{1}{N_{normal}+N_{babv}} \right)VR}{{2C}_{alveolar}}$$

We can then form the ratio of the two tensions:

$$\frac{T_{baby}}{T_{normal}}=\frac{\frac{\left( \frac{1}{N_{normal}+N_{babv}} \right)VR}{{2C}_{alveolar}}}{\frac{\left( \frac{1}{N_{normal}+N_{babv}} \right)VR}{{2C}_{alveolar}}}=1$$

$$\Longrightarrow T_{baby}=T_{normal}$$

Hence, the tension in each alveolus of the two compartments is the same. This derivation assumed that the alveoli in both the normal and baby compartments are being inflated to the same volume. If this requirement on the volume is relaxed, then one can derive a different relation between the tensions. For example, if $V_{normal}=\alpha V$ and $V_{baby}=\left( 1-\alpha\right)V$ where $0<\alpha<1$, then

$$T_{normal}=\frac{\alpha VR}{{N_{normal}C}_{alveolar}}$$

and

$$T_{baby}=\frac{(1-\alpha)VR}{{N_{babv}C}_{alveolar}}$$

Then the ratio of tensions become:

$$\frac{T_{baby}}{T_{normal}}=\frac{(1-\alpha)N_{normal}}{{\alpha N}_{babv}}$$

$$\Longrightarrow T_{baby}=\left( \frac{(1-\alpha)N_{normal}}{{\alpha N}_{babv}} \right)T_{normal}$$

As $\alpha\longrightarrow0$, *i.e*., most of the volume goes to the baby compartment, then $T_{baby}\gg T_{normal}$. As $\alpha\longrightarrow1$, *i.e*., most of the volume goes to the normal compartment, then $T_{baby}\longrightarrow0$. Hence, the relationship between the tensions in the baby and normal compartments is dependent on the number of alveoli in each compartment and how the incoming tidal volume is split between the two compartments.

**Part 3: Rationale for Lung Unit Radius Estimate**

A. The two key variables defining tension are the pressure $\left( P \right)$ within the balloon and the radius $\left( R \right)$ of the balloon, the first of which can be directly measured in clinical practice and the other only estimated. This part of the supplement outlines the rationale underpinning that estimation. In broad outline, the value of $R$ for the spherical lung expressed in clinical terms derives from its absolute volume:

$$V=FRC+C\cdot PEEP+ V_{T}$$

With the resting volume $(FRC)$ estimated as described in the main text, $R$ of a sphere at either the macro (lung) or micro (subunit) scales is determined from its corresponding absolute volume as: $R=\left( \frac{3V}{4\pi} \right)^{1/3}$. With this value for the radius, we can estimate the tension using Laplace’s equation: $T=\frac{1}{2}P\cdot R$. Furthermore, the surface area of the sphere can be calculated using $A=4\pi R^{2}$.

Although the strain increment associated with V_T_ is often thought of as $\frac{\Delta V}{V_{rest}}$, perhaps a better estimation involves the surface areas ∆A_s_ to the A_s_ at rest, while the increments of T & A_s_ comprise the incremental elastic energy stored within the membrane during inflation.

B. Relating the airspace pressures (P) and tidal volumes (V_T_) that clinicians use to these individual components, P values for PEEP and P_plat_ are measured relative to their shared zero reference at the lung’s unstressed resting volume (FRC), and V_T_ is referenced to the volume at PEEP. In this simplified mathematical model that ignores collapse, the ‘zero *volume*’ origins of R for the volumes that correspond to FRC, PEEP and P_plat_ are identical. However, the ‘zero *pressure*’ reference point (PEEP=0 cmH_2_O) occurs at the unstressed FRC volume. In principle, the value of R for the spherical lung is derived from its absolute volume (V=FRC+ PEEPxC+V_T_ ). With resting volume estimated, R of the sphere on any scale is determined from its corresponding absolute volume (V) as: R= (3V/4π)^1/3^. This R can then be used to estimate the ‘membrane’ tensions (T = PR/2) and ‘membrane’ areas, A_s_ = 4πR^2^ of interest.

Note that in this model, T for any lung subunit at FRC is calculated to be nil when no PEEP is applied because measured P is zero when measured from FRC, even though subunit *volume* is a function of the P-defined ‘unstressed’ V_rest_. (Like the other volume components, FRC does, of course, have an unmeasured true transmural distending pressure.) For each of the ‘n’ subunits that comprise a baby lung, the relevant unstressed resting volume is: FRC_su_ = V_rest_ /n. Furthermore, for each of ‘n’ subunits, the absolute volume, V = (FRC + PEEPxC +[DPxC])/n.

**Part 4: Area-Volume Ratio with Changing Radius**

For a sphere of radius $R$, we examine how changes in $R$ change its surface area and volume. Using the formulas for area and volume of a sphere, we have:

$$V=\frac{4}{3}\pi R^{3} \Longrightarrow dV=4\pi R^{2}dR$$

$$A=4\pi R^{2} \Longrightarrow dA=8\pi RdR$$

Therefore,

$$\frac{dA}{dV}=\frac{8\pi RdR}{4\pi R^{2}dR}=\frac{2}{R}$$

or

$$dA=\left( \frac{2}{R} \right)dV$$

Hence, $\frac{dA}{dV}\leq1 \Longrightarrow\frac{2}{R}\leq1 \Longrightarrow R\geq2$. On the other hand, if $\frac{dA}{dV}>1$, then $R<1$. This result carries over the increments of the area and volume. In particular,

$$\frac{\Delta A}{\Delta V}<1 (R>2)$$

$$\frac{\Delta A}{\Delta V}>1 (R<2)$$

where we have defined: $\Delta F\equiv F\left( R+\Delta R \right)-F(R)$.
